# An Application of Eye Movement Parameters Collected from Mass Market Devices for the Estimation of a Text Comprehension

**DOI:** 10.16910/jemr.16.2.1

**Published:** 2023-03-20

**Authors:** Ksenia Babanova, Arsen Revazov, Konstantin Chernozatonskiy, Andrey Pikunov, Victor Anisimov

**Affiliations:** Oken Technologies, Inc., USA; Lomonosov MSU, Faculty of Biology, Russia

**Keywords:** Eye movement, eye tracking, gaze fixations, reading, comprehension, gaze detection algorithms

## Abstract

The growing interest in evaluating the reader's comprehension leads to the search for new methods that allow such estimation in real-time (or pseudo-real-time). This can be used for more effective educational processes and to adopt textual content for various purposes. The present study used the Oken Reader eye-tracking application (60 Hz) for mass-market devices to assess reading comprehension processes. Twenty-three (23) respondents aged between 19 and 31 (mean = 24. 5, SE = 1. 4, 65% female) participated in the study. The mean, mu, and sigma parameters differed significantly depending on the level of text comprehension. This result indicates the possibility of using mass-market devices with eye-tracking technology to assess comprehension in reading. Furthermore, the study's results confirm the possibility of searching the correlations between physiological indicators such as eye movements and comprehension.

## Introduction

Comprehension as a cognitive function has attracted researchers’ attention in different areas of science and education for a long time (6; Ferreira, 2019). It is often defined as a complex representation of human mental activity, can be objectively characterized by parameters of cognitive state, and relates to neural activity of the central nervous system. Comprehension could be presented as a construction of an associative structure in our mind (12; 13). Comprehension is a complex cognitive process. Information obtained from perceived words allows us to imagine, reveal and decompose the heroes' internal essence, relations, and communications, as well as the hidden meanings in the text and scenes (11; 20).

Readers with higher level of comprehension (“good comprehenders”) read more frequently than poor ones; thus, they are less likely to encounter low-frequency linguistic stimuli, and they are skilled at encoding linguistic input into structures that can be quickly and easily retrieved from the long-term memory when needed (7) and demonstrate more excellent parafoveal preview benefits (22). Literacy skill in adulthood is associated with systematic differences in global and lexically driven eye-movement control during reading (24; 32; 31; 26; 22).

Eye movements are associated with working memory functioning (7; 16), selective attention processes (15), executive control (16) in reading, global passage difficulty (24) and cognitive workload (21). The effectiveness and speed of visual information processing can be reflected in such a parameter of eye movements as the fixation duration.

Reading comprehension/understanding is particularly interesting to educational practice (12; 18). Real-time (or pseudo-real-time) comprehension assessment would enable determination, whereas the situation requires the reader’s or teacher’s adjusting actions. It could also reduce the time costs and the length of low-efficiency periods when a reader demonstrates insufficient attention and level of comprehension.

Which tool could make this possible? Currently, there are expectations for physiological and behavioral signals that can be measured objectively, such as cardiovascular autonomic, electrodermal, respiratory, muscular (including facial muscles), electroencephalographic, and eye movements parameters (5; 27). Among various physiological approaches (5; 27), eye tracking is one of the most precise methods to assess online comprehension demand in reading (24; Binkley et al., 2013; 23; 2; 36; 33; 29; 17; Shaner & Donmoyer, 2022). However, most existing eye trackers are adapted to the scientific laboratory environment to a greater extent than for application in educational processes. Consequently, in the actual study, we proposed using an eye-tracking technology for determining text comprehension with only one mass-market device widely used by students and many others (IPad).

Eye movement parameters and fixation duration in reading are influenced by attention processes (21), word frequency (32; 35), predictability (31; 28; 9), word length, parafoveal preview (26; 28), word position in the sentence, context (34) and individual differences such as working memory span (10; 16), literacy (22), language experience, and general reasoning (7).

Stationary eye trackers, eye trackers with higher sampling rates, or high-resolution eye trackers are used to assess perception and (mis)comprehension. For example, EyeTech™ Digital Systems VT2 at 80 Hz sampling rate, SMI RED250 250 Hz (2), SREyelink2 (24), SR EyeLink 1000 Hz ( 23; 28; 22; 34; 9). There are few pieces of evidence about applying simple, affordable, and “less laboratory” eye tracker models for this purpose. However, there are now several solutions on the market that allow recording eye movements using inexpensive devices, and eye tracking technology is already present in user devices.

When the sample rate of an eye tracking device is relatively low (e.g., 60 Hz), the fixation duration can be calculated with an extensive dispersal. Distributional analysis of fixation durations introduced by Staub (32) in eye tracking may be particularly successful for this case, along with mean and median estimation. The natural distribution of fixation durations may be fitted as ex-Gaussian, a combination of so-called ‘normal’ or Gaussian distribution and exponential tail (32; 8). Three parameters describe the ex-Gaussian distribution; two parameters correspond to the normal component (μ, the mean, and σ, the standard deviation), and a single exponential parameter (τ) that is influenced by different factors in reading (32).

The mu (μ) parameter is independent of individual differences such as age and literacy level, correlated across the tasks (scene viewing, reading, searching tasks) for each individual, and can be predicted by working memory span in scene viewing, but not in reading (16; 22). At the same time, word predictability influences the μ-parameter (31; 28). Adults, compared to children, showed an effect of contextual constraint on all the gaze duration quantiles (34). High-frequency words were associated with a decrease in both μ and τ ex-Gaussian parameters for the first fixation duration compared to low-frequency words and shorter gaze duration; low-frequency words were associated with a more significant proportion of refixations (35). Working memory span is related to the size of the tail of the fixation duration distribution in the opposite way: a higher working memory span was associated with fewer long fixations and long saccades in reading (16). Lower literacy is associated with an increased τ parameter (22). Activation of a right inferior frontal gyrus, associated in particular with syntactic and semantic integration, has a negative correlation (0. 4 < Spearman ρ <0. 5) with σ and τ (10). Older age and lower literacy levels are predictors of increased σ, i. e. the more considerable variability of fixation duration during reading (22).

Based on these data, we proposed that eye movements distributional analysis can provide insights into the mechanisms underlying text comprehension and, thus, prospective for future development of eye-tracking-based tools for these online assessment processes.

Using the most popular comprehension control, i.e., 4-choice questions, the general hypothesis was that reading comprehension using a 60 Hz eye-tracking system allows evaluating the reading comprehension level based on fixation duration.

Thereby, the study focuses on the application of eye movement registration methods for reading texts from the screen of a digital device. The authors in this study had several main research questions: 1) evaluation and demonstration of modern methods of eye movements registration integrated into user devices (on the example of the tool developed by the team), 2) application of the method in assessment of perception and furthermore comprehension of the text, 3) demonstration of a limitations of the technology itself and of the methodology of research issues relevant to the modern remote format of working with text-based educational materials.

The present work is of interest for the field of education and focuses on the process of perception and comprehension of an information, which is typical for the modern educational format.

## Methods

The experiment was conducted using an eye-tracking application on a mass-market device (IPad). Respondents read augmented and unaugmented texts and answered the questions. Reading order and augments were manipulated to examine eye-movements dependency on comprehension level.

### Oken Reader

The team developed an iPad application called Oken Reader with ARkit-based eye-tracking technology. The iOS gadgets starting in 2019 have ARkit technology on the board and can be used with the app. In this experiment, the texts were presented on the iPad Pro 11 inches screen.

Raw eye movement data were stored on a secure server, where ML algorithms can also be applied. Eye movement events were extracted from the raw track using the machine learning Catboost algorithm. It has benefits like the built-in overfitting detector and close to optimal hyperparameters initialization. Eye-movements precision of 0. 97 was achieved based on manual event detection by an expert and another precisely verified eye tracker (see 1).

**Figure 1.**
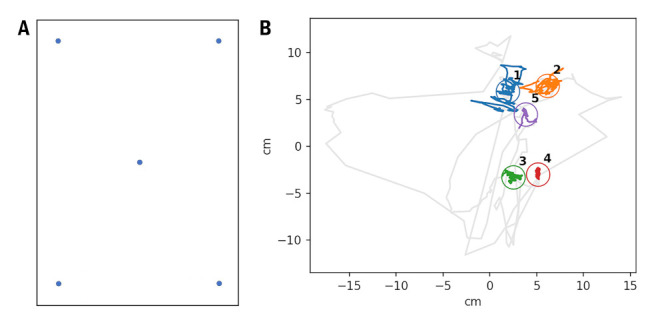
Oken Reader precision and accuracy. A. IPad screenshot with five calibration dots. B. An example of a raw gaze track for one of the respondents with identified calibration dots.

The software has a user-friendly, convenient interface for text presentation and implements a record of the respondent’s eye tracking data. The method's precision was assessed using a calibration pattern that included five fixation dots in the corners of the screen and its center. Based on several records, the precision was estimated as 0. 8 degrees, which is reasonably high. At the same time, the accuracy of the method is low. Eye tracks on the stimuli have a high level of instability and angular distortions (Figure 1). Our previous work also reported this problem (1).

The main focus of the research was on the fixation duration and their distribution. Since the primary sampling rate was 60 Hz, the fixation duration has an error of 16.6 ms.

### Participants

Twenty-three (23) respondents participated in the study, aged between 19 and 31 years (mean = 24. 5, SE = 1. 4, 65% female). All respondents were right-handed, native speakers and had a normal or corrected-to-normal vision. They all reported normal or good general physical and mental states in the pre-test questionnaire. Reading speed was also tested before the experiment. The group mean was 175. 9±10. 2 words/minute (min = 113, max = 282).

Accepted bioethical standards conducted the study. Respondents gave informed voluntary consent and received a monetary reward for their participation.

### Materials

Three text stimuli were used in the study. The first text was about the working of the heart described in physio-logical style (*heart*), the second was a narrative about a woodpecker (*woodpecker*) and its ability to withstand head traumas due to biomechanical features of its anatomy, and the third one was a text in the area of pediatric medicine (*pediatrics*).

Table 1 shows the linguistic characteristics of the texts used. Flesch-Kincaid, SMOG (Simple Measure of Gobbledygook), Coleman–Liau, and Automatic readability indices were adapted for the Russian language. The percentage of long and low-frequency words and the total number of words were also analyzed. All the texts are hard to read and suitable for an adult audience. Although *woodpecker* text is more accessible in terms of readability indices, it contains 19% of low-frequency words.

Additional information was added to each of the texts, to some extent revealing the topic of the text and/or visualizing the primary terms (e.g., heart valves and their names were shown). The addition of preliminary information was intended to increase the respondent's awareness of the text topic and/or update relevant knowledge. This approach allows for a more significant number of correct answers and also increases the variability of the number of correct answers received by each respondent. At the same time, the additional materials demonstration was conducted in advance, i.e., before text reading. Thus, an influence on the reading procedure was not expected, but a change in the level of text comprehension.

Three types of supplements were used. The type of additional information was selected according to the content of each text. The first type (pediatrics) was constructed according to the methodology described by Martin et al. (18) and contained infographics. This facilitated the perception of the information, as it allowed schematically presenting the text's content. The second (heart) was the video content with illustrations and explanatory diagrams. The third (woodpecker) had annotation, which indicated the critical essence embodied in the text.

### Design

**Figure 2.**
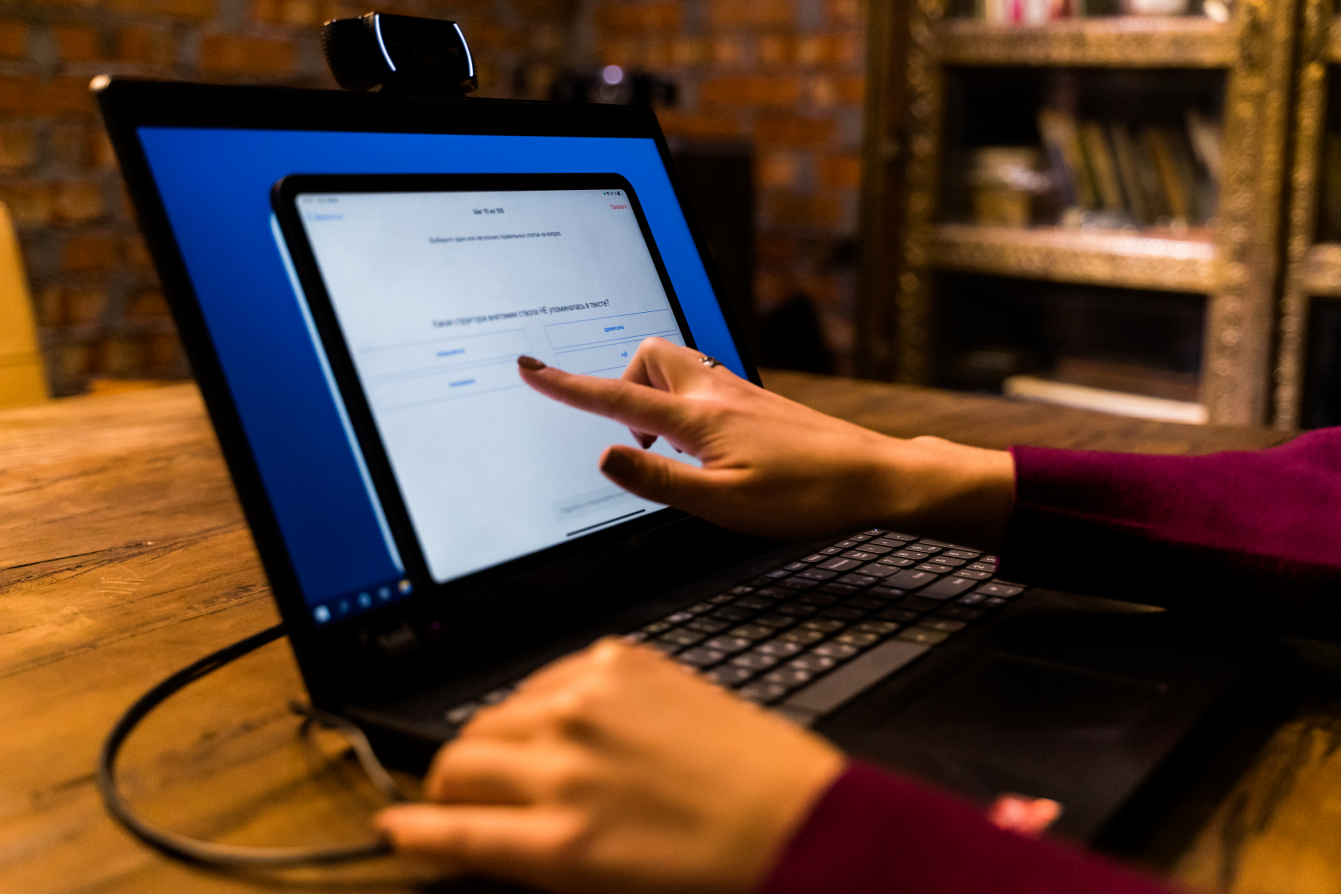
Reading and choosing answers to questions in the developed Oken Reader application on the iPad. The choice was made by tapping the button with one of the answer options.

The texts were comfortably presented on the iPad screen from the respondents' eyes, approximately 50 cm. The iPad was fixed on a stand (laptop screen, Figure 2) in a stable position. It was the solution from a previous series of experiments (1) with simultaneous recording of eye movement data with Tobii 4c connected to a laptop (control). The respondent’s head was not fixed, and movements were unrestricted. Reading speed was self-paced, as respondents were using page-by-page scrolling or tapping.

Respondents read texts in two forms. The first was texts supplemented with additional nonverbal information as described in the Materials section. The second included only text. Augmented and unaugmented texts were shown in a pseudo-random order, so each respondent encountered both text forms. After viewing the preliminary information, the respondent read the exact text as in the case without the supplement. Thus, a particular respondent read a given text either with or without a preface, and there was no re-reading of the exact text and no habituation. After each text, respondents answered three comprehension questions (with 4-answer options) by choosing possible answer with buttons (Figure 2).

### Procedure

All the procedures were carried out with RStudio Build 554. First, fixations were compared regarding associations between eye movement strategy during reading and the answers to the specific questions. For this purpose, the distributional analysis described by Staub (32) was used, assuming that the natural distribution of fixation duration may be fitted as an ex-Gaussian and vincentiles approach specified by (3).

The distribution parameters, including μ, σ, and τ, were obtained using retimes package (19). Then, linear mixed-effects models (LMMs) were performed with the Lme4 (4) and lmerTest (14) packages to estimate the measure and respondent random effects and the fixed effect of the number of correct answers.

## Results

Fixation duration analysis was conducted for the four groups based on the number of correct responses: all three were correct (all true, AT), two correct (most true, MT), one correct (most false, MF), and no correct responses (all false, AF).

### Distributional analysis

**Figure 3.**
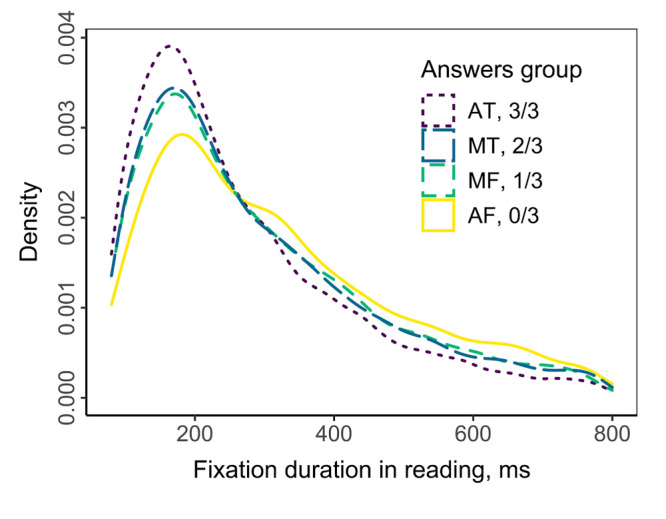
Density function of fixation durations during reading in respect with the subsequent number of correct answers

An analysis was performed including all fixations with the first pass and re-readings. Fixations associated with the moving between pages, with the choosing response to questions during the presentation of questions, as well as fixations outside the screen area were excluded from the analysis. Fixation data were filtered in the 80-800 ms range. The density distribution of fixation duration is shown in Figure 3. Data are presented for 68 texts; one measure was excluded from the analysis due to poor quality. As shown in Figure 3, the decreasing number of correct answers reflects the shift of distribution to the right and the right part of distribution has inversion compared with the left part. This reflects that when respondent failed to give a correct answer, he/she experiences more long fixations and less short fixations in reading.

Test data internal consistency (Cronbach’s α) was acceptable for the test results (α = 0. 71). Question difficulty was calculated as the proportion of participants with the correct answer (P). The mean difficulty was MP = 0. 657 (SE = 0. 042), ranging between P = 0. 33 and P = 1. We found no decrease in the difficulty of the items with the small Cohen’s d = 0. 21. Meanwhile for the text supplemented with video content the Cohen’s d = 0. 55 indicated medium-size effect. The final distribution of respondents across response groups was not equal. Six (26%) respondents answered within one group, twelve (52%) respondents answered within two groups, and only five (22%) respondents answered within three different study groups, but not simultaneously in AT and AF groups.

Thus, despite the manipulation of additional information, as well as the selection of rather complex texts, the respondents tended towards correct or incorrect responses. Unfortunately, this result does not allow repeated measures ANOVA or mixed ANOVA because some of the necessary data points need to be included. Therefore, the first part of the analysis presents mainly descriptive statistics of the distribution of fixation duration as a function of the number of correct answers.

To test whether there is a relationship between the duration of fixations and the level of understanding of the text (the number of correct responses), a distribution analysis was performed (Fig. 3). An ex-Gaussian approximation was built for each text of each respondent, and its parameters were determined, as well as other parameters presented in Table 2.

**Table 2.** Ex-Gaussian parameters by answers group for fixation durations in reading.

Group	Correct answers	Measures number	Fixations number	Fixations by text	Mean	Median	Mode	μ	σ	τ
AT	3/3	25	11581	472 (208)	274 (41)	236 (41)	179 (35)	152 (39)	84 (33)	122 (7)
MT	2/3	23	11466	507 (292)	294 (39)	252 (40)	180 (54)	167 (38)	99 (25)	127 (8)
MF	1/3	12	7338	621 (291)	298 (42)	260 (48)	181 (54)	174 (44)	103 (32)	124 (8)
AF	0/3	8	3497	453 (284)	318 (19)	282 (24)	179 (35)	190 (21)	116 (14)	127 (7)
p-value	-	-	-	0.535	0.005	0.005	0.0476^a^	0.008	0.004	0.1

Note: Standard errors (SE) are given in parentheses. Statistical comparison between groups was conducted using one-way ANOVA with dFn = 1 and dFd = 66. a – the distribution violated normal distribution, Kruskal-Wallis test result.

**Figure 4.**
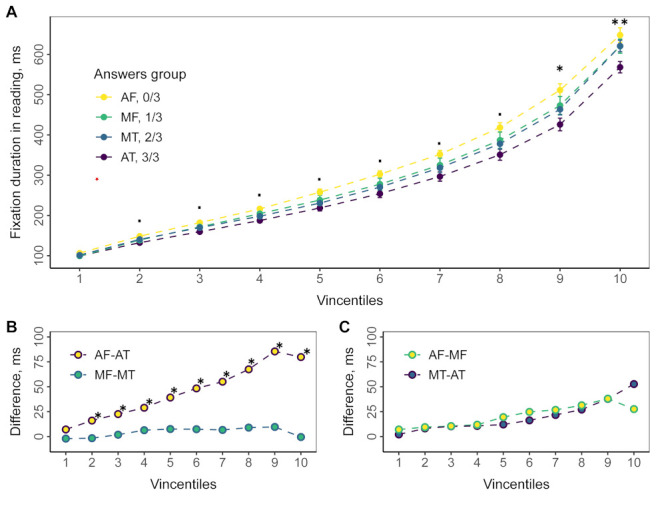
Empirical vincentiles for fixation durations in reading. Every vincentile consists of 10% of ordered fixation duration data. Points represent mean value averaged across all participants; error bars reflect standard errors. Colors stand for answers groups. A. Group empirical vincentiles. Significance level based on the one-way ANOVA: ∙ p < 0. 1, * p < 0. 05, ** p < 0. 01. B, C. Difference in mean fixation duration for answers groups. Significance level based on the pair-wised t-test: * p < 0. 05.

A one-factor ANOVA or Kruskal-Wallis test was used to compare between all groups. A preliminary test for normality was performed using the Shapiro-Wilk test (Table 2, p-value). Vincentiles with one-way ANOVA results are shown on Figure 4. Each vincentile represents the 10% of the data; the circle represents its mean. The filtered data was ordered from the lowest to the highest value to obtain the bins. The first 10% of the ordered data is form the first bin, the second 10% from second bin, etc. Thus, we have ten equal bins with the same number of observations (fixations). Note that the number of fixations related to each measure can be not multiple of 10. Thus, we excluded the highest 1-9 fixations not to violate the equality of bins. Alternatively, these fixations could be added to the 10^th^ quantile. However, this would not change the statistical results in this quantile. To get vincentiles for the group of respondents, the vincentiles for each were obtained and then averaged (3).

Analysis of 10% of the bins revealed statistically significant differences depending on the number of correct answers to comprehension questions in bins 9 and 10. The pairwise comparison revealed differences between the AF and AT groups (Figure 4, B). No statistically significant differences were found between the other groups. This means the main effect relates to the long durations.

### Mixed effects modeling

LMMs are known for their ability to account for missing data, so they are the best way to analyze data in an uneven distribution of respondents across correct response groups.

Linear mixed-effects models were run on the fixation durations data to estimate the effect of correct responses and other factors. The model is presented in Table 3. The model was aimed to obtain respondent, text, and supplement effects and fixation duration in reading in predicting text comprehension. In addition, to account for the respondent’s data in a more explicit form, the reading speed was measured and used, obtained before the experiment as a fixed effect. Formula was “number of correct answers ~ fixation duration + individual reading speed + (1 | text) + (1 | supplemented)”.

**Table 3.** Results of the LMM model to test the effect of fixation duration in reading, individual reading speed, the text read, and preliminary information adjustment (supplement) on the number of correct responses to comprehension questions after reading..

Fixed effects				
Independent	Predictor	Estimate	t-value	P
Number of correct answers	Intercept	2 (0. 3)	6. 187	0. 0129
	Fixation duration	-5. 736 *10^-4^ (2. 904 * 10^-5^)	-19. 755	< 0. 001
	Reading speed	1. 043 *10^-3^ (1. 026 *10^-4^)	10. 165	< 0. 001
Random effects				
Independent	Groups	Name	Variance	
Number of correct answers	Text	Intercept	0. 27570 (0. 5251)	
	Supplemented	Intercept	0. 02996 (0. 1731)	
	Residual		0. 757551(0. 8704)	

Note: Standard errors (SE) are given in parentheses.

The present model demonstrates that an individual’s reading speed is also a predictor of the number of correct answers and the form of the fixation duration distribution in reading. Moreover, this result indicates that the reading item should also be considered when discussing reading comprehension based on the fixations characteristics.

## Discussion

Fixation duration reflects the processes of visual perception span and information processing. According to the natural reading model, the focus of visual attention is controlled mainly based on semantic analysis results. The success of the semantic analysis of the read text can be reflected in a decrease in the time of decoding information and a reduction in the time to correct decoding errors. Thus, the success of semantic analysis (reading comprehension) will decrease the duration of fixations on subsequent reading fragments (24).

The relationship between fixation duration in reading and the number of correct responses to comprehension questions in the actual study indicates the continuous nature of comprehension. It is based on the consequential eye movements associated with the perception and processing of information in working memory. The groups with different numbers of correct responses had statistically significant differences in ex-Gaussian parameters and other characteristics of fixation duration distribution. However, the most pronounced differences were between AT and AF groups when all responses were correct or incorrect. The decrease in μ and σ could probably reflect the repeated effective lexical processing leading to a correct response. Along with this, the tendency (p = 0. 1) of decreased τ component in AT could be explained by better attentional and control processes, facilitating perceptual and contextual integration (16; 22; 34).

In addition to the analysis of the ex-Gaussian parameters, the results of distributional analysis (Fig. 3) could also be interpreted in terms of the lexical processing, described by Reingold with colleagues (26, see Fig. 6d). The overlapping nature of the density curves in different answers group can be explained by varying degrees of lexical processing success (especially for low-frequency words) and other success in parafoveal previewing. This can also be supported by the previous studies on word predictability, contextual constraint, and misperceptions (31; 9) and the studies of low-frequency preview conditions (28). Individual reading speed, markers for the development of reading skills, or/and language experience should also be considered when discussing text comprehension, as it reflects an individual’s disposition to make correct lexical decisions.

Our results also suggest that text supplements, particularly in the video format, could introduce the context, basic vocabulary, and the meaning of unknown words by additional visual cues, thereby increasing the likelihood of short-time *recall* in a test and overall text *comprehension*. However, the natural effect of introducing additional information before reading has yet to be accurately investigated in future work.

One might also hypothesize that readers with eye tracking patterns similar to AT group patterns better understand the text, memorize information, and thus perform better academic scores and results. Meanwhile, fewer correct responses do not necessarily reflect worse comprehension but probably different degrees of guessing luck. A combination of the methods used in the study with predicting response confidence (36) sheds light on whether this claim is valid. Otherwise, even poor lexical decisions and inadequate text comprehension in group AF cannot be a marker of inadequate understanding of the text matter or even principal mechanisms of phenomena contained in texts. In this context, *summarizing* can be an additional tool for the assessment of the overall level of comprehension and interpretation of corresponding eye movement patterns. In particular, it was shown that selective attention processes influence an individual reading strategy. Some readers who are presumably, more sensitive to topic-relevant information use a selective reading strategy (15).

For practice purposes, the eye tracking approach we demonstrated in our work can be implemented in online comprehension detection for reading tasks with certain reservations. This advantage becomes more valuable with the development of eye tracking solutions integrated into modern mass-market electronic devices. Furthermore, assessing the eye movement patterns in their dynamics can formalize and objectify hidden cognitive processes.

Eye movements (as a physiological activity reflecting the processes of attention, concentration, and cognitive activity in the perception of information in general) may indicate such a complex mental process as comprehension. The present study shows the potential possibility of using physiological indicators that are recorded not *a posteriori* but directly in the process of perceiving verbal information to assess the comprehension of the content being read for educational and other purposes, for example, for assessing the user's condition when he is involved in reading certain documents, articles, license agreements, etc. using a mass-marked device with low-sampling rate eye tracking technology.

### Limitations

However, despite its broad phenomenological nature, the study has several limitations. First, the number of respondents could have been higher in terms of an excess and balanced number of correct or incorrect answers: respondents commonly tend to have more correct or incorrect answers. This can be solved by recruiting more respondents and cutting off part of the answers of one of the classes (correct or incorrect) to equalize comparison groups. The number of the sample should have been more significant to account for individual differences and their nature.

Secondly, the number of texts and questions is limited to avoid readers' tiredness and the overwhelming effects of information mixing, as all texts were relatively difficult to read. We could also notice some difficulties associated with it (1) aligning the range of preliminary information that should help to understand the main text (for example, it is difficult to unify additional text for biomechanics and medicine), (2) producing the questions of “equal coverage” for different texts, that were expertly created. These points influence both the experiment design balance and comprehension estimation.

Thirdly, the study demonstrates a physiological approach in assessing comprehension and, in fact, the case study of the application of eye tracking technology embedded in modern individual mass market devices.

### Ethics and Conflict of Interest

The authors declare that the article's contents agree with the ethics described in http://biblio.unibe.ch/portale/elibrary/BOP/jemr/ethics.html and that there is no conflict of interest regarding the publication of this paper.

### Acknowledgments

We thank Oken Technologies, Inc. company based on which the research was carried out.
